# Independent Preharvest Applications of Methyl Jasmonate and Chitosan Elicit Differential Upregulation of Defense-Related Genes with Reduced Incidence of Gray Mold Decay during Postharvest Storage of *Fragaria chiloensis* Fruit

**DOI:** 10.3390/ijms18071420

**Published:** 2017-07-03

**Authors:** Gabriela M. Saavedra, Eugenio Sanfuentes, Pablo M. Figueroa, Carlos R. Figueroa

**Affiliations:** 1 Master Program in Forest Sciences, Faculty of Forest Sciences, University of Concepción, Concepción 4070386, Chile; gabisaa@gmail.com; 2 Forest Pathology Laboratory, Faculty of Forest Sciences, University of Concepción, Concepción 4070386, Chile; esanfuen@udec.cl; 3 Phytohormone Research Laboratory, Institute of Biological Sciences, University of Talca, Talca 3465548, Chile; pabfigueroa@utalca.cl

**Keywords:** *Botrytis cinerea*, chitinases, β-1,3-glucanases, elicitors, polygalacturonase-inhibiting proteins, priming, strawberry

## Abstract

The Chilean strawberry (*Fragaria chiloensis*) fruit has interesting organoleptic properties, but its postharvest life is affected by gray mold decay caused by *Botrytis cinerea*. The effect of preharvest applications of methyl jasmonate (MeJA) or chitosan on the molecular defense-related responses and protection against gray mold decay were investigated in Chilean strawberry fruit during postharvest storage. Specifically, we inoculated harvested fruit with *B. cinerea* spores and studied the expression of genes encoding for the pathogenesis-related (PR) proteins β-1,3-glucanases (*FcBG2-1*, *FcBG2-2* and *FcBG2-3*) and chitinases (*FcCHI2-2* and *FcCHI3-1*), and for polygalacturonase inhibiting proteins (*FcPGIP1* and *FcPGIP2*) at 0, 2, 24, 48, and 72 h post inoculation (hpi). Remarkably, MeJA- and chitosan-treated fruit exhibited a lower incidence of *B. cinerea* infection than the control-treated at 48 and 72 hpi. At the molecular level, both are efficient elicitors for priming in *F. chiloensis* fruit since we observed an upregulation of the *FcBG2-1*, *FcBG2-3*, *FcPGIP1,* and *FcPGIP2* at 0 hpi. Moreover, a chitosan-mediated upregulation of *FcPGIP*s at early times post inoculation (2–24 hpi) and MeJA upregulated *FcBG*s (24–72 hpi) and *FcPGIP1* at later times could contribute to reduce *B. cinerea* incidence by differential upregulation of defense genes. We concluded that preharvest applications of MeJA or chitosan had a long-lasting effect on the reduction of *B. cinerea* incidence during postharvest as well as an enhancer effect on the induction of *PR* and *PGIP* gene expression.

## 1. Introduction

The Chilean strawberry (*Fragaria chiloensis* (L.) Mill.) fruit has emerged as a new alternative for the global market of berries. It has interesting organoleptic properties, presenting a particular white color, good taste, and an intense aroma. Besides having the potential to become a new berry fruit to broaden fruit offerings, it also has the potential to be used for genetic *Fragaria* × *ananassa* Duch. improvement due to the interesting traits found in genotypes recently characterized in our germplasm collection [1]. Being a non-climacteric fruit, strawberries do not ripen postharvest and therefore must be harvested at the nearly full-ripe stage [2]. Despite its favorable organoleptic properties, the *F. chiloensis* fruit has a short shelf life due to a fast softening rate [3] and a susceptibility to infection by necrotrophic fungi during postharvest storage [4]. *Botrytis cinerea* Pers. Ex. Fr, a causal agent of gray mold disease, is a widely distributed necrotrophic fungus, and is one of the most destructive strawberry diseases worldwide. It can cause yield losses of up to 25% for non-protected strawberries [5] in spite of a certain degree of tolerance to fungal infection found in Chilean populations of *F. chiloensis* [6]. Traditionally, the control of postharvest gray mold decay relies mainly on the use of synthetic fungicides; however, long term monitoring of the fungicide resistance of *B. cinerea* initiated in French wine-growing regions has revealed the appearance of strains with cross resistance to chemically unrelated fungicides [7]. Due to the increasing resistance of fungal pathogens to fungicides and the growing concern of consumers over chemical residues, it is necessary to study alternative ways to better control postharvest diseases [8] through the reinforcement of natural plant defense systems.

The perception of necrotrophic pathogens, such as *B. cinerea* or their associated signals by specific receptors in plant tissues, triggers the mitogen-activated protein kinase (MAPK) cascades and activates jasmonates (JAs)- and ethylene-dependent signaling pathways involving the activation of specific transcription factors [9]. These can trigger the expression of a large number of genes and finally, mount a defense response against the fungus. Various proteins induced by necrotrophic pathogens are collectively referred to as pathogenesis-related proteins (PRs), which are produced by the host plant, but are mainly induced under pathogen infection [10]. The main PR classes cause damage to the structures of the microbial plant pathogens. PR1 and PR5 interact with the fungi plasma membrane, whereas β-1,3-glucanases (BGs; PR2) and chitinases (CHIs; PR3, PR4, PR8, and PR11) hydrolyze β-1,3-glucans and chitin, respectively, which are cell wall components in most of the higher fungi [11]. Recently, coding genes for PR5 and PR10 have been characterized in *F. chiloensis* [12]; nevertheless, BGs and CHIs are the most abundant classes of strawberry PRs with hydrolytic activity identified thus far [13]. On the other hand, among the microbe-detecting molecules that are used by the plant immune system to activate the primary response against pathogens, are the polygalacturonase-inhibiting proteins (PGIPs) [14]. PGIPs have been reported to play a defensive role in strawberry fruit since transgenic lines with a constitutive expression of the *FaPGIP* gene showed a lower susceptible phenotype against *B. cinerea* than the untransformed lines [15]. Until now, no information on BGs-, CHIs-, and PGIPs-encoding genes has been reported in *F. chiloensis*.

The defense-related phytohormone methyl jasmonate (MeJA) and the biological elicitor chitosan have been described as inductors of resistance during postharvest storage in many plant species. As a plant-signaling molecule, MeJA has been reported to enhance disease resistance in various fruit during postharvest storage [16,17]. On the other hand, the biopolymer and biological elicitor chitosan is another promising treatment due to its natural character, antifungal activity, and elicitation of defense responses in plant tissues [18]. Preharvest and postharvest treatments with chitosan and its derivatives tend to suppress storage fruit rot in many commodities, including strawberries [19], sweet cherries [20], and table grapes [21]. Several studies have demonstrated that chitosan is an exogenous elicitor of host defense responses, including the accumulation of CHIs, BGs and phenolic compounds, induced lignification, synthesis of phytoalexins by the infected host tissue, and inhibition of host tissue maceration enzymes [22]. It has also been observed that chitosan had antifungal activity against *B. cinerea* when tested in vitro [23]. Previously, we showed that independent preharvest applications of MeJA and chitosan to *F. chiloensis* fruit also led to an increments in anthocyanin, antioxidant capacity, and lignin accumulation along with a delay in postharvest decay triggered by both elicitors [4].

**Scheme 1:**
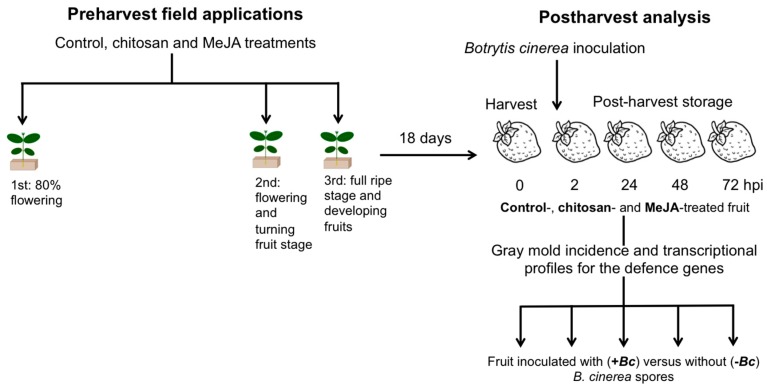
Schematic research methodology. Three preharvest applications of 250 μmol·L^−1^ methyl jasmonate (MeJA), 1.5% (*w*/*v*) chitosan or water (control) were performed during flowering, fruit development, and ripening of *Fragaria chiloensis*. Eighteen days later, ripe fruit were harvested and subjected to inoculation with (+*Bc*) or without (–*Bc*) *Botrytis cinerea* spores. Fruit were stored at room temperature and the incidence of gray mold and the transcriptional profiles of defense genes were analyzed at 0, 2, 24, 48, and 72 h post inoculation (hpi). For experimental details, see the Materials and Methods Section.

Previous studies indicate that priming defenses by natural compounds could be a cost-effective strategy to protect harvested strawberry fruit from *B. cinerea* infection and consequently quality loss. This is a novel comparative molecular study reporting the effects of preharvest applications of MeJA or chitosan on fruit during postharvest storage. Thus, to investigate the defense associated-molecular response to the preharvest applications of the MeJA and chitosan in strawberry fruit in depth, we analyzed and contrasted the expression levels of the BGs-, CHIs-, and PGIPs-encoding genes during postharvest *F. chiloensis* fruit in response to *B. cinerea* inoculation (Scheme 1). We showed that both preharvest applications had a long-lasting effect that reduced *B. cinerea* incidence during the postharvest storage of Chilean strawberry fruit.

## 2. Results and Discussion

### 2.1. Postharvest Decay Incidence of Gray Mold in F. chiloensis Fruit Is Lower in MeJA Than Chitosan-Treated Fruit 

To study the response of gray mold infected fruit to either MeJA or chitosan elicitors, we inoculated with (+*Bc*) or without (–*Bc*) *B. cinerea* spores (Scheme 1). It should be noted that all fruit used for the different treatments were at the same ripening stage at the beginning of the inoculation test. The fruit were harvested at the ripe stage as defined by Figueroa et al. [3]. At this stage, there is the highest soluble solids concentration (SSC)/titratable acidity (TA) ratio in this fruit [24], which could increase susceptibility to pathogen infections. It is important to mention that despite a lack of strong evidence whether MeJA or chitosan could change the ripening rate of strawberry fruit in planta, we measured the beginning of the postharvest storage (0 h) fruit ripening parameters such as fruit firmness, weight, and the skin color parameter a* (which denotes red coloration in strawberry fruit) and we did not observe differences between the values of the different treatments (Table S1). At the end of the storage period (72 h), no differences in weight and the above-mentioned color parameter between treatments were found, although chitosan-treated fruit showed a greater fruit firmness than the control- and MeJA-treated fruit (Table S1). In addition, we had previously reported that the SSC/TA ratio and fruit skin color did not show any important differences regarding MeJA or chitosan applications during the postharvest storage of *F. chiloensis* fruit [4]. In this study, we focused on the specific molecular plant responses to *B. cinerea* infection in preharvest MeJA- or chitosan-treated fruit.

**Figure 1:**
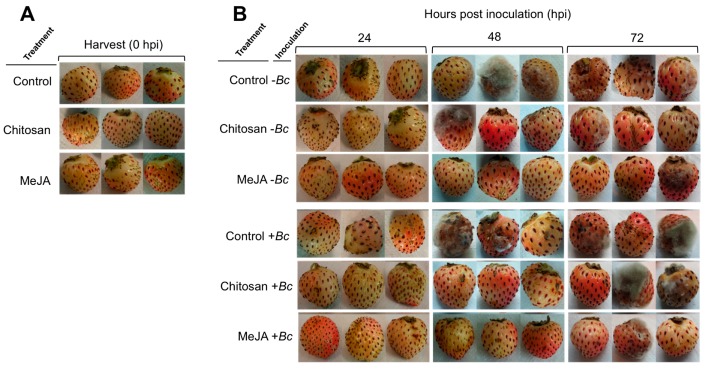
Symptoms and signs evolution in *Fragaria chiloensis* fruit treated with methyl jasmonate (MeJA) and chitosan infected with *Botrytis cinerea* at different hours post inoculation (hpi). Representative photographs of three fruit from the total for each treatment (control, chitosan, and MeJA) and inoculation (with (+*Bc*) or without (–*Bc*) *B. cinerea* spores) at harvest (**A**), and from 24 to 72 hpi (**B**). For experimental details see the Materials and Methods Section.

A representative progression of gray mold infection in fruit at different time-points (0, 24, 48, and 72 h post inoculation; hpi) during postharvest storage is shown in Figure 1. In –*Bc* fruit, the MeJA-treated fruit showed a lower gray mold incidence (%) from 24 to 72 hpi (0, 0, and 33% for 24, 48, and 72 hpi, respectively) than the chitosan-treated ones (33, 67, and 67% for 24, 48, and 72 hpi, respectively) (Table 1), which indicates that MeJA was better than chitosan at protecting against gray mold infection. Moreover, MeJA-treated fruit exhibited a healthier physical appearance than the chitosan-treated ones, including +*Bc* fruit at 48 and 72 hpi (Figure 1). Furthermore, the fungus incidence for MeJA-treated fruit was lower (33%) than that observed in chitosan-treated ones (67%) at 72 hpi either in the +*Bc* and –*Bc* fruit (Table 1). Thus, we suggest that MeJA could act as a more effective antifungal compound against *B. cinerea* than chitosan, given that MeJA-treated fruit showed a lower incidence value than the chitosan-treated ones at 48 and 72 hpi either in –*Bc* and +*Bc* fruit.

It was remarkable to note that the +*Bc* fruit previously treated with either MeJA or chitosan showed a lower incidence of gray mold than the +*Bc* control at 48 and 72 hpi (Figure 1; Table 1). At the end of storage (72 hpi), MeJA-treated fruit showed 33% of disease incidence against 67% and 100% for chitosan-treated, and control fruit, respectively (Table 1). Furthermore, it has been shown that the application of jasmonic acid (JA) and MeJA can reduce the decay incidence for several kinds of fruit. Postharvest 10 µM JA treatment reduced green mold (*Penicillum digitatum* (Pers. Fr.) Sacc.) incidence in orange fruit, correlating with an increase in the expression of the β-1,3-endoglucanase-encoding gene [25]. MeJA treatment not only upregulated *PR* expression, but also promoted higher phenylalanine ammonia-lyase activity and increased total phenolics, flavonoids, and anthocyanins levels in several berries, including the Chinese bayberry and Chilean strawberry [4,26,27]. In Chinese bayberries, MeJA treatment induced resistance against *Penicillium citrinum* Thom by priming defense responses through the upregulation of the hydrogen peroxide burst and the enhancement of defense-related protein levels and the antimicrobial compound content [28]. In this sense, the lower gray mold incidence observed in MeJA-treated fruit may be due to the overall beneficial effects of this compound on fruit. In turn, applications of chemical elicitors including chitosan, have been reported to induce the activities of defensive enzymes and/or upregulate *PR*, which is closely linked to induced disease resistance and reduced disease severity in strawberries [29].

### 2.2. Priming Effect of MeJA and Chitosan on Chilean Strawberry Fruit

**Figure 2:**
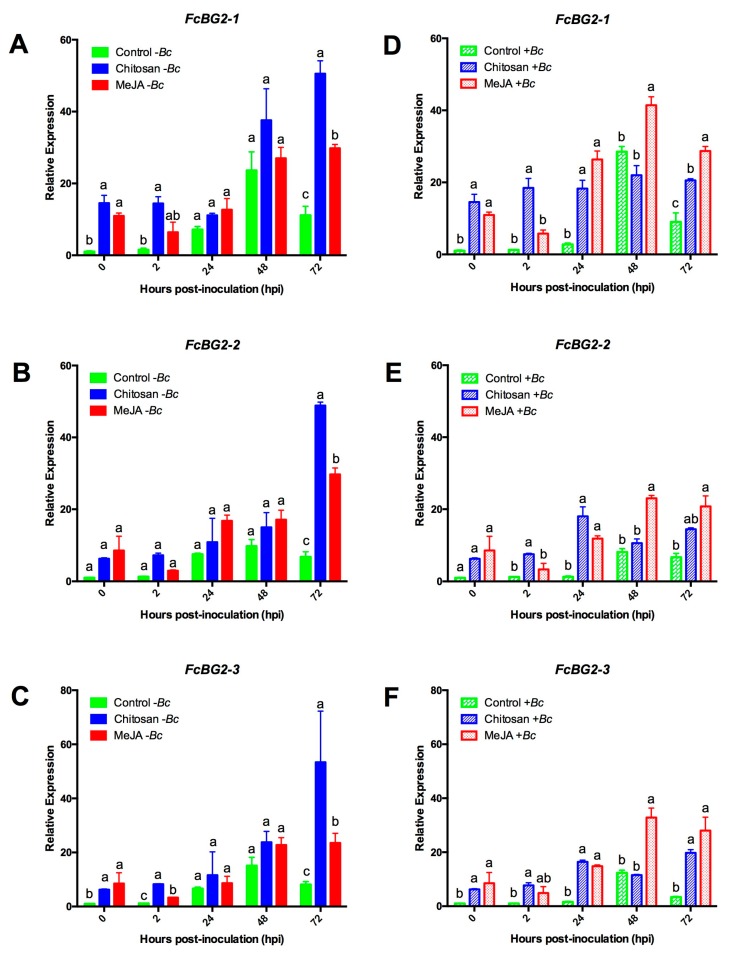
Expression profiles for β-1,3-glucanases-encoding genes (*BG*s) in *Fragaria chiloensis* fruit treated with methyl jasmonate (MeJA) or chitosan at different hours post inoculation (hpi) with or without *Botrytis cinerea* spores*.* Changes in the relative expression of *FcBG2-1*, *FcBG2-2*, and *FcBG2-3* in fruit inoculated without (–*Bc*) (**A**–**C**) and with (+*Bc*) *B. cinerea* spores (**D**–**F**), respectively, at 0, 2, 24, 48, and 72 hpi. Data were analyzed by ANOVA test and differences between means ± standard error (SE) (*n* = 3) were determined using the Tukey test. Different letters indicate significant differences between treatments (*p* < 0.05) for each hpi. For experimental details, see the Materials and Methods Section.

**Figure 3:**
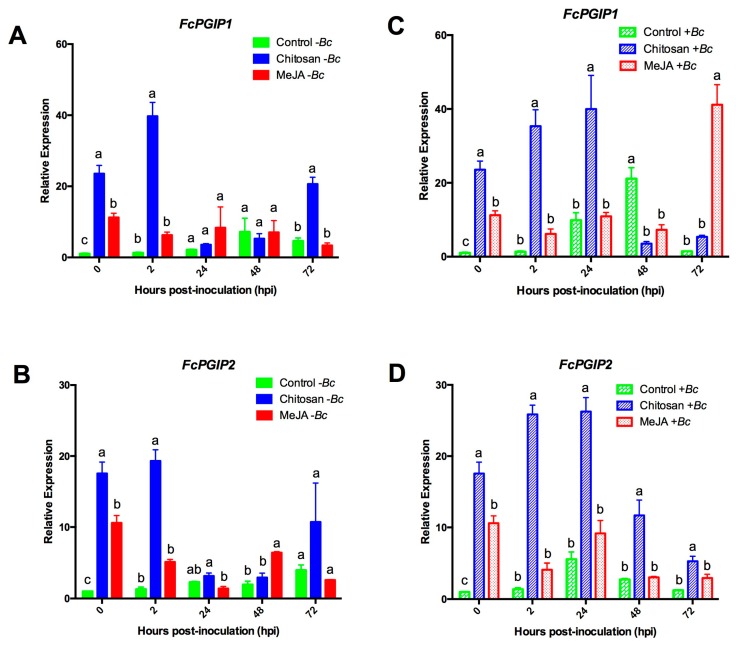
Expression profiles for polygalacturonase inhibiting protein-encoding genes (*PGIP*s) in *Fragaria chiloensis* fruit treated with methyl jasmonate (MeJA) or chitosan from 0 to 72 h post inoculation (hpi) with or without *Botrytis cinerea* spores*.* Changes in relative expression of *FcPGIP1* and *FcPGIP2* in fruit inoculated without (–*Bc*) (**A**,**B**) and with (+*Bc*) *B. cinerea* spores (**C**,**D**), respectively, at 0, 2, 24, 48, and 72 hpi. Data were analyzed by ANOVA test and differences between means ± SE (*n* = 3) were determined using the Tukey test. Different letters indicate significant differences between treatments (*p* < 0.05) for each hpi. For experimental details see the Materials and Methods Section.

Aside from physical and chemical constitutive barriers, plants have developed a defense line that can be induced after the detection of phytopathogenic microorganisms via plant immune receptors [30]. There are two types of induced resistance in plants: systemic acquired resistance (SAR) and induced systemic resistance (ISR). Both can induce defenses that confer long-lasting protection to plants against a broad spectrum of microorganisms, and are mediated by phytohormones such as salicylic acid, JAs, and ethylene [31]. An induced resistance does not directly produce defense responses in plant; however, it invokes an alert state to deal rapidly and efficiently with an attack. This phenomenon is known as the priming effect [31,32]. In this study, we observed the upregulation of the defense *FcBG2-1, FcBG2-3, FcPGIP1*, and *FcPGIP2* genes triggered by the application of MeJA or chitosan at the beginning of fruit storage (0 hpi), and before the *B. cinerea* inoculation, suggesting that both compounds were efficient elicitors for priming in Chilean strawberry fruit (Figure 2 and Figure 3). It was important to consider whether the plants from which the fruit came from received their last application 18 days before harvest (Scheme 1), thus reaffirming that these elicitors had long-lasting effects in the induction of defense-related genes during postharvest in *F. chiloensis* fruit.

### 2.3. Differential Upregulation of Defense-Related Genes by MeJA or Chitosan during Postharvest Storage of F. chiloensis Fruit 

**Scheme 2:**
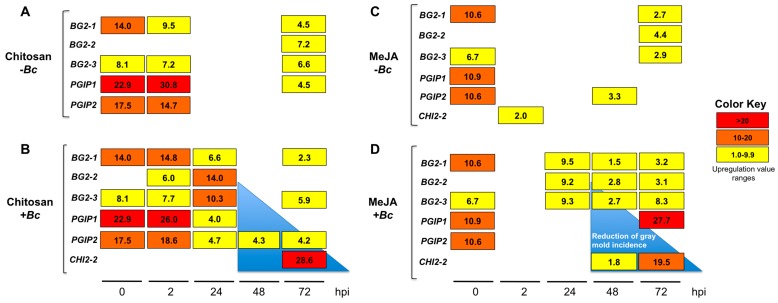
Temporal sequence of upregulation of *PR* and *PGIP* genes and gray mold incidence in chitosan- and MeJA-treated *Fragaria chiloensis* fruit at different hours post inoculation (hpi). Upregulation pattern of defense genes in Chitosan –*Bc* (**A**), Chitosan +*Bc* (**B**), MeJA –*Bc* (**C**), and MeJA +*Bc* (**D**) treatments at 0, 2, 24, 48, and 72 h post inoculation (hpi). Values in each box indicate the fold-change of upregulation for each gene with respect to the value of the control treatment according to the color key shown. Blue triangles represent the reduction of gray mold incidence observed in Chitosan +*Bc* and MeJA +*Bc* with respect to Control +*Bc* treatment. For experimental details see the Materials and Methods Section. *PR* genes: *BG2-1*, β-1,3-glucanase 2-1; *BG2-2*, β-1,3-glucanase 2-2; *BG2-3*, β-1,3-glucanase 2-3; *CHI2-2*, chitinase 2-2; *CHI3-1*, chitinase 3-1. *PGIP* genes: *PGIP1*, polygalacturonase inhibiting protein 1; *PGIP2*, polygalacturonase inhibiting protein 2. MeJA: methyl jasmonate.

To study changes of expression for several defense-related genes triggered either by elicitors, fungus, or both, we performed a gene expression analysis by RT-qPCR of the +*Bc* and –*Bc* fruit either in the control, chitosan, or MeJA treatments (Scheme 1). Overall, the transcriptional profiles revealed an upregulation of *PR BG*s (*FcBG2-1*, *FcBG2-2*, and *FcBG2-3*; Table 2) and of defense-related *PGIP*s (*FcPGIP1* and *FcPGIP2*; Table 2) for chitosan or MeJA treatments in +*Bc* fruit at 2, 24, and 72 hpi (Figure 2D–F and Figure 3C,D, and Scheme 2B,D). 

**Figure 4:**
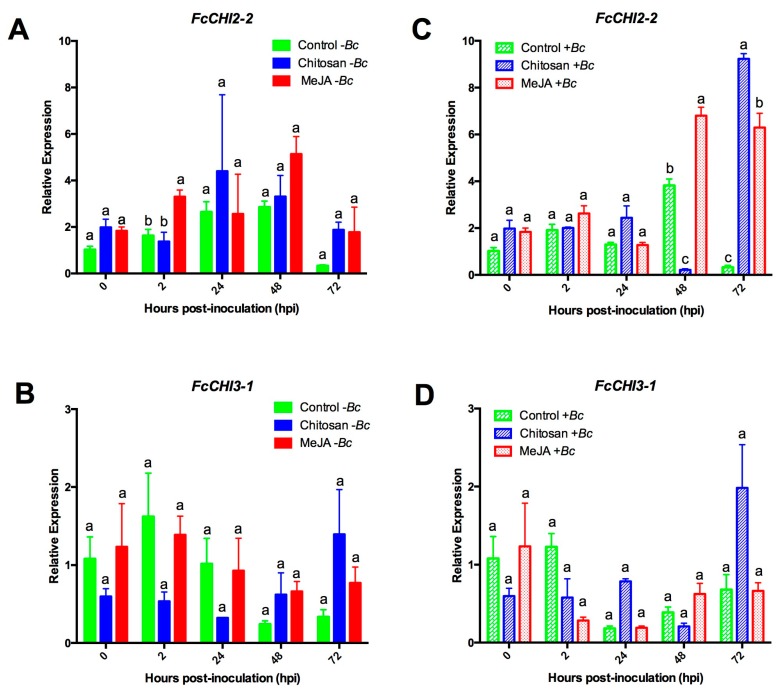
Expression profiles of chitinase-encoding genes (*CHI*s) in *Fragaria chiloensis* fruit treated with methyl jasmonate (MeJA) or chitosan at different hours post inoculation (hpi) with or without *Botrytis cinerea* spores*.* Changes in relative expression of *FcCHI2-2* and *FcCHI3-1* in fruit inoculated without (–*Bc*) (**A**,**B**) and with (+*Bc*) *B. cinerea* spores (**C**,**D**), respectively, at 0, 2, 24, 48, and 72 hpi. Data were analyzed by ANOVA test and differences between means ± SE (*n* = 3) were determined using the Tukey test. Different letters indicate significant differences between treatments (*p* < 0.05) for each hpi. For experimental details, see the Materials and Methods Section.

We found that MeJA upregulated *BG, CHI*, and *PGIP* genes with different patterns and levels in Chilean strawberry fruit during *B. cinerea* infection (Figure 2, Figure 3 and Figure 4). The induction of some of these genes was rapid and transient regardless of the presence of *B. cinerea* spores (+*Bc* or –*Bc*; i.e., *FcPGIP2*; Figure 3B,D). Other genes (i.e., *FcBG2-1*, *FcBG2-2*, *FcBG2-3*, and *FcCHI2-2*; Figure 2D–F and Figure 4C) were induced later and increased over time after inoculation with *B. cinerea* spores, whereas others were upregulated at the end of the storage period (i.e., *FcPGIP1*; Figure 3C). On the other hand, we observed that chitosan upregulation of the *BG, CHI*, and *PGIP* genes was different with respect to that observed with MeJA. Remarkably, the chitosan-treated fruit exhibited the highest upregulation of *FcBG* genes at 72 hpi in –*Bc* fruit (Figure 2A–C), and an early upregulation of *FcPGIP* genes from 0 to 24 hpi in +*Bc* fruit (Figure 3C,D). Additionally, chitosan-treated fruit showed an upregulation of *FcCHI2-2* gene in +*Bc* fruit at 72 hpi (Figure 4C). 

#### 2.3.1. Expression Profiles for β-1,3-glucanases (*BG*s) and Chitinases (*CHI*s) Genes

The expression levels for *FcBG*s increased only slightly in the control –*Bc* and +*Bc* fruit until 48 hpi, and then decreased to 72 hpi (Figure 2). MeJA and chitosan upregulated the *FcBG* genes only at 72 hpi in –*Bc* fruit, with greater values in chitosan-treated fruit than MeJA-treated ones (Figure 2A–C). Particularly, the expression of *FcBG2-1* had a differential pattern in control –*Bc* fruit when compared to those of *FcBG2-2* and *FcBG2-3* (Figure 2A–C). It showed a peak at 48 hpi in contrast to the more regular expression pattern observed for *FcBG2-2/2-3* genes (Figure 2A–C; Table S2). In this sense, Pombo et al. [33] reported different patterns for two strawberry *BG* genes analyzed both in control and UV-C-treated fruit. The increase in the *FcBG2-1* expression level at 48 h in –*Bc* fruit (Figure 2A) could be related to a differential expression regulation to which this gene may be subjected. The presence of endogenous *B. cinerea* inoculum in –*Bc* fruit (Table 1) could be upregulating strawberry *BG* genes differently, according to differences in response elements in their promoter regions.

When comparing *BG* expression levels between –*Bc* and +*Bc* fruit, we noted that they were downregulated in chitosan-treated fruit inoculated with *B. cinerea* spores at specific times after inoculation. Specifically, *BG2-1* at 48 and 72 hpi, and in *BG2-2* and *BG2-3* at 72 hpi significantly decreased (Table S3). Regarding the +*Bc* fruit, the expression profiles triggered by both elicitors had a differential upregulation pattern compared with those observed in –*Bc* fruit. In chitosan-treated fruit, the expression pattern of the different *FcBG* genes was similar being higher at 2, 24, and 72 hpi than the control fruit, whereas MeJA upregulated *FcBG* genes steadily at 24, 48, and 72 hpi (Figure 2D–F). In this sense, chitosan upregulated *FcBG2-1* 14.8-, 6.6-, and 2.3-fold; *FcBG2-2* 6-, 14-, and 2.2-fold; and *FcBG2-3* 7.7-, 10.3-, and 5.9-fold with respect to the control at 2, 24, and 72 hpi, respectively (Figure 2D–F, Table S4). In the case of MeJA, this compound upregulated *FcBG2-1* 9.5-, 1.5-, and 3.2-fold; *FcBG2-2* 9.2-, 2.8-, and 3.1-fold; and *FcBG2-3* 9.3-, 2.7-, and 8.3-fold with respect to the control at 24, 48, and 72 hpi, respectively (Figure 2D–F, Table S4). Moreover, in +*Bc* fruit, it was noted that the expression level of *FcBG* genes was higher in MeJA- than in the chitosan-treated fruit at 48 and 72 hpi (Figure 2D–F). In agreement, the orthologous *BG* genes *FaBG2-1* and *FaBG2-3* were found upregulated upon the hemibiotrophic fungi *Colletotrichum fragariae* Brooks and *Colletotrichum acutatum* Simmonds infections, and by UV-C treatment in *F.* × *ananassa* fruit [33,35,36,37].

In the case of *CHI*s*, FcCHI2-2* exhibited remarkable upregulation at 72 hpi in +*Bc* fruit with both elicitors with respect to the control +*Bc* at the same time point, being a 19.5 and 28.6-fold increase with MeJA and chitosan treatments, respectively (Figure 4C, Table S4). Only MeJA-treated fruit exhibited an upregulation at 48 hpi in comparison with the control (Figure 4C). For –*Bc* fruit, *FcCHI2-2* showed upregulation only at 2 hpi in MeJA-treated fruit (Figure 4A). In *F.* × *ananassa* fruit the orthologous *CHI* gene *FaCHI2-2* was also induced by *C. fragariae*, *C. acutatum*, and *B. cinerea* infections, and was upregulated in UV-C-treated fruit [33,38,39] as seen in the case of the *BG* genes previously mentioned. Meanwhile, *FcCHI3-1* did not show any significant change in its expression level with both the MeJA and chitosan treatments, and *B. cinerea* inoculation (Figure 4B,D).

In bean plants, the chitinase *PvChit1* gene was significantly upregulated during the necrotrophic fungus *Sclerontinia sclerotiorum* (Lib.) de Bary infection (6–72 hpi) previously treated with MeJA via foliar application [40]. This may have contributed to the observed increased resistance in bean plants, whereas the over-expression of chitinase in transgenic cucumber plants has been reported to reduce or retard the development of disease symptoms after fungal attack in studies involving *B. cinerea* infection [41]. On the other hand, the *CHI*- and *BG*-related transcript levels increased rapidly in tobacco cell suspensions reaching a maximum around 24 h after the addition of a cell wall preparation from *Phytophthora parasitica* Dastur var. *nicotianae* or MeJA as elicitors [42]. Particularly, the orthologous *CHI* gene *FaCHI3-1* was described as expressed constitutively at low levels in *F.* × *ananassa* leaves [43], but with an increased expression in fruit immediately after UV-C treatment [33]. However, we observed that *FcCHI3-1* was not regulated either by elicitor treatments or by *B. cinerea* inoculation during the postharvest storage of *F. chiloensis* fruit (Figure 4B,D).

#### 2.3.2. Expression Profiles for the Polygalacturonase Inhibiting Proteins (*PGIP*s) Genes

For *FcPGIP1* and *FcPGIP2* genes, chitosan-treated fruit showed the highest values of upregulation both in –*Bc* and +*Bc* fruit at 0 and 2 hpi (14.7 to 30.8-fold increase in relation to the controls at the respective time points; Scheme 2, Table S4); however, chitosan upregulated both *PGIP*s at 24 hpi (Figure 3C,D) only in the +*Bc* fruit. In the case of the *FcPGIP2* gene, chitosan upregulated its expression also at 48 and 72 hpi, albeit to a minor extent with regard to earlier time points (Figure 3D). MeJA-treated fruit showed an upregulation of *FcPGIP*s genes at 0 hpi (Figure 3) and only of *FcPGIP1* in +*Bc* fruit at 72 hpi with a 27.7-fold increase with respect to the control (Figure 3C, Table S2).

The observed distinctive gene expression patterns between *PGIP*s genes could be explained by variations on the spatio-temporal expression under elicitors and/or biotic stress [44]. In strawberries, seven *FaPGIP* variants from five strawberry cultivars were identified and upon fruit inoculation with *B. cinerea*, all cultivars exhibited a significant induction in *FaPGIP* expression [45,46]. In our study, we also observed an induction of both *PGIP*s by *B. cinerea* alone (Figure 3C,D). Oliveira et al. [40] also showed that the bean *PvPGIP* family was expressed and their transcripts (*PvPGIP1* and *PvPGIP2*) underwent a stronger accumulation upon MeJA pre-treatment in bean plants following *S. sclerotiorum* infection.

#### 2.3.3. MeJA and Chitosan Upregulate *FcBG*s and *FcPGIP*s which Correlate with Reduction of *B. cinerea* Incidence in *F. Chiloensis* Fruit

This research was a comparative study on the activation of defense-related genes either in MeJA or chitosan applications in fleshy fruit. *B. cinerea* triggered the upregulation of defense genes *FcBG2-1, FcBG2-2*, *FcBG2-3*, *FcPGIP1*, and *FcCHI2-2* at 48 hpi in the control fruit (Figure 2D–F, Figure 3C, and Figure 4C), although only in *FcBG2-1, FcBG2-3*, *FcPGIP1,* and *FcCHI2-2* were these increases significantly higher with respect to 0, 2, and 24 hpi (Figure 2D,F, Figure 3C and Figure 4C, Table S2). However, the application of chitosan or MeJA set forward the upregulation of *FcBG* at least 24 h before (Figure 2D–F) and *FcPGIP* genes up to earlier times post inoculation (0–24 hpi) and upregulated all these genes over time (48–72 hpi) (Figure 2D–F and Figure 3C,D, Scheme 2B,D), which likely helped to reinforce the fruit defense mechanisms against *B. cinerea* infection and reduce the incidence of the pathogen (Table 1, Scheme 2B,D). In this sense, *FcBG* genes were upregulated in MeJA-treated fruit at almost all times post infection, but differed from the chitosan-treated ones since *FcBG*s upregulation was higher and more steady at late times (48 and 72 hpi) (Scheme 2D). In contrast, chitosan upregulated *FcPGIP* genes mainly at early times (0–24 hpi) (Scheme 2B), whereas MeJA did not upregulate these genes except for *FcPGIP1* at 72 hpi (Figure 3C). In this sense, chitosan and MeJA preharvest applications could be complemented for the activation of *PR*s and *PGIP*s during fruit postharvest, being two synergistic elicitors for the reinforcement of plant defense mechanisms.

## 3. Materials and Methods

### 3.1. Plant Material, Treatments, and Fruit Quality Assessments

Ripe fruit were harvested from *F. chiloensis* plants grown in a commercial orchard located in Purén, Araucanía Region, Chile (latitude 38°04′S; 73°14′W) and treated with preharvest applications of 250 μmol·L^−1^ MeJA (Sigma–Aldrich, St. Louis, MO, USA) and 1.5% (*w*/*v*) chitosan (IONA Ltd., Santiago, Chile) at pH 4.3 using 0.05% (*v*/*v*) Tween-20 as a surfactant in both solutions. Distilled water plus 0.05% (*v*/*v*) Tween-20 was used as a control. One hundred and thirty plants were used per treatment under the same agronomic management. Crop were sprayed three times at different developmental stages: the first at 80% flowering (80% full bloom and 20% flower buds without fruit); the second at flowering (100% full bloom) and turning fruit stage (C3 stage according to Figueroa et al. [3]); and the third at full ripe fruit stage (C4 stage according to Figueroa et al. [3]) and developing fruit. Postharvest evaluations were made in fruit harvested after 18 days of the third application (Scheme 1). The harvested fruit were immediately transported to the laboratory under refrigerated conditions. Fruit for the experiment were selected based on uniform size, shape, color, and surface damage. Twenty-four fruit from each treatment were maintained at 22 °C and approximately 60% relative humidity for 72 h and analyzed for fruit quality assessments. At 0 and 72 h of storage, 12 fruit from each treatment were weighed, and the skin color and fruit firmness were recorded as previously reported in [4].

### 3.2. Botrytis cinerea Isolates and Aggressiveness Assay

The *B. cinerea* strain pool used in this study was isolated from the botrytis-infected flowers and leaves of *F.* × *ananassa*. Infected material was kept in a humidity chamber for 48 h before spores were taken, resuspended in sterile water, placed in a Petri plate containing 2% (*w*/*v*) potato dextrose agar (PDA) and incubated at 20 °C. Cultures were replicated in the same culture media to obtain pure cultures over 12 days. Two isolates from flowers and one from leaves were obtained. For the aggressiveness assay, host material was prepared and inoculated. Chilean strawberry leaf discs of 1.5 mm diameter were cut and placed on the adaxial side of the agar medium in Petri dishes. The inoculation was performed by adding an inoculum suspension (10^6^ spores/mL) on discs and the aggressiveness of each isolate was evaluated on five discs in two replications. The intensity of sporulation on each disc was assessed based on a visual severity scale [47] for selecting the strain used for further assays. According to this scale, the leaf isolate with a value of 6.1 was selected amongst the other from flowers that had values of 5.7 and 4.9. For inoculum production, cultures were placed in a PDA medium and incubated for a 12 h photoperiod at 22 °C. After 10 days, spores were collected with sterile distilled water (SDW). The spore suspension was filtered through sterile cheese cloth and diluted to 10^6^ spores/ml using SDW. Final suspension and inoculation control (SDW) were adjusted with 0.02% (*v*/*v*) Tween-20. 

### 3.3. Botrytis cinerea Inoculations in Postharvest Conditions

All MeJA-, chitosan-treated, and control fruit were superficially disinfested by immersion in a 1.5% (*v*/*v*) sodium hypochlorite solution for 1 min, washed three times with SDW, and dried on absorbent paper. The fruit from preharvest treatments were inoculated by placing 20 µL of the spore suspension (+*Bc* fruit) or SDW (–*Bc* fruit) (Scheme 1) with a 50 µL sterile syringe at the equator of each fruit as previously reported in [48]. The +*Bc* and –*Bc* fruit were incubated at 22 °C with approximately 60% relative humidity for 0, 2, 24, 48, and 72 h post inoculation (hpi). For storage, one layer of fruit was packaged in perforated plastic containers (12.5 cm width, 12.5 cm depth, and 4.5 cm height).

### 3.4. Analysis of Incidence and Gene Expression Analysis 

Incidence and gene expression analysis were recorded at 0, 2, 24, 48, and 72 hpi of fruit infected with *B. cinerea*. At each sampling time point, three replicates of six fruit each were employed for the different analyses. Gray mold incidence was measured as a percentage of infected fruit (pathogen sporulation on the fruit surface) for each treatment and time. Total RNA was extracted from receptacle tissue (including achenes) around the inoculation site (approximately 1 cm^3^) following the cetyltrimethylammonium bromide (CTAB) method [49] with modifications. Three biological replicates were used for each treatment. Samples were purified using RNA mini-columns (RNeasy Plus mini kit, Qiagen, Hilden, Germany). All RNA samples were quantified by fluorescence (Qubit 2.0, ThermoFisher Scientific, Waltham, MA, USA) and integrity was confirmed on agarose gels. Reverse transcription for cDNA synthesis was performed using 1 µg of total RNA (RevertAid H minus First Strand cDNA Synthesis Kit, Thermo Scientific, Helsinki, Finland). The transcriptional profile of seven genes encoding different PR and PGIP proteins for each treatment and time was analyzed by quantitative reverse transcription PCR (RT-qPCR) in a Real-Time PCR System (PikoReal, Thermo Scientific, Vantaa, Finland). Specifically, we analyzed the expression levels of β-1,3-glucanases (*BG2-1*, *BG2-2*, and *BG2-3*), chitinases (*CHI2-2* and *CHI3-1*) and polygalacturonase-inhibiting proteins (*PGIP1* and *PGIP2*) (Table 2). Glyceraldehyde 3-phosphate dehydrogenase (*GAPDH*) was used as the reference housekeeping gene [27]. Specific primer sequences were designed using Primer3 software [50] directly from sequences of *F.* × *ananassa* available on GenBank [34] (Table 2). Primers were evaluated by end-point PCR and all generated single PCR products. Five serial dilutions were used to evaluate the efficiency of every primer used on real time PCR. The amplification reactions were performed in triplicate per sample (biological replicate) using the SYBR FAST qPCR kit (KAPA Biosystems, Boston, MA, USA) as per the manufacturer’s recommendations and the thermal profile was: 95 °C for 10 min; 40 cycles of 95 °C for 15 s, 60 °C for 15 s, and 72 °C for 15 s, and final melting curve of 95 °C for 15 s, 60 °C for 1 min, and 95 °C for 15 s. Each reaction was performed in triplicate (technical replicate), and a no template control was included in each run. Fluorescence was measured at the end of each extension step. The relative expression levels corresponded to the mean of three biological replicates normalized against the mean calculated for the expression level of the housekeeping gene. Control fruit from 0 day were used as the calibrator sample and assigned a nominal value of 1. The expression levels were calculated according to the 2^−∆∆*C*t^ method [51] and expressed in arbitrary units.

### 3.5. Statistical Analysis

All experiments were performed using a completely randomized design, with the main factors being treatments (control, MeJA, and chitosan), *Botrytis* inoculation (with botrytis spores (+*Bc*) or not (–*Bc*)) and hours post inoculation (0, 2, 24, 48, and 72 hpi). Analysis of variance (ANOVA) was used to compare the means of each gene expression value between treatments in each hpi using SAS/STAT^®^ version 9.1.3 software (Cary, NC, USA). Differences were considered statistically significant at *p* < 0.05 (Tukey test) and were indicated with different letters (Figure 2, Figure 3 and Figure 4, Table S4, Scheme 2). Additionally, we performed multiple comparison analysis between all mean values in +*Bc* and –*Bc* fruit per each gene (Tables S2,3) using GraphPad Prism^®^ version 6.00 software (La Jolla, CA, USA).

## 4. Conclusions

Induced defense responses in plants are extremely complex, and more than one type of defense can be engaged simultaneously. Based on the obtained expression patterns for the defense genes, we concluded that the preharvest foliar applications of MeJA and chitosan had a strong enhancer effect on the induction of defense pathways by means of upregulation of *PR*s and *PGIP*s, which correlated to an observed reduced incidence of *B. cinerea* in Chilean strawberry fruit. This protective effect, triggered by each elicitor, could be mediated by a primed state that led to the upregulation of specific defense genes on a temporal basis, since we observed long-term effects of MeJA and chitosan on defense-related systems. Our results showed that preharvest treatments of *F. chiloensis* fruit with MeJA or chitosan would be an environmentally friendly alternative to chemical fungicide to delay the onset of *B. cinerea* infection during the strawberry postharvest.

## Figures and Tables

**Table 1:** Incidence (%) ^1^ of gray mold in the control-, chitosan-, and MeJA-treated *Fragaria chiloensis* fruit inoculated with (+*Bc*) or without (–*Bc*) *Botrytis cinerea* spores during postharvest storage.

Treatment ^2^	Inoculation ^2^	Hours Post Inoculation (hpi) ^3^				
		0	2	24	48	72
Control	–*Bc*	0	0	0	67	67
Chitosan	–*Bc*	0	0	33	67	67
MeJA	–*Bc*	0	0	0	0	33
Control	+*Bc*	0	0	33	100	100
Chitosan	+*Bc*	0	0	0	67	67
MeJA	+*Bc*	0	0	33	0	33

^1^ Visual evaluation of *F. chiloensis* fruit with *B. cinerea* mycelium (*n* = 6); ^2^ For experimental details see the Materials and Methods Section; ^3^ Representative photographs of the *B. cinerea* infection progression are shown in Figure 1.

**Table 2:** Strawberry defense genes and primers sequences used for the gene expression analysis ^1^.

Gene ^2^	Accession No. ^1^	Protein Family ^3^	Strawberry Gene ID or Standardized Gene Name ^3^	Forward Primer	Reverse Primer	Amplicon Size (pb)	Efficiency (%)
*BG2-1*	AY170375	PR2	*FaBG2-1*	CTAAATATCTTCTTCCTGCCATA	AATGTTGTATCTATTGCTGTT	101	103
*BG2-2*	AY989818	PR2	*FaBG2-2*	ACCGGGACTCCCAAGAGACCAAATG	TGTGAGCCTGCACTAGCCAAAGGTG	162	86
*BG2-3*	AY989819	PR2	*FaBG2-3*	TCCGAGAGTGGTTGGCCATCTGAAG	TCCATTTGGTCTCTTCGGAGTCCCG	117	101
*CHI2-2*	AF320111	PR3	*FaCHI2-2*	GCACAACAGGTGATGTTGC	GTAATGACGTCGTGGCTTGA	183	100
*CHI3-1*	AF134347	PR8	*FaCHI3-1*	AGGTCTTCTTAGGACTCCCTG	CTTGGACCAAAGCATGACACCGCC	134	81
*PGIP1*	EU117215	PGIP	*FrveA6 PGIP*	TGCTAGAATTCGATCTGTCCAAGG	ATTATCCAATTGGGTCAACTGCTC	113	103
*PGIP2*	EU117213	PGIP	*FaPGIP-1.8*	TCCTCATGGAAATCCGACGCCGAC	ACCTGTGAGATTGGGGAGCTTGCG	189	101

^1^ The primers for *BG2*s, *PGIP*s, and *CHI*s genes were designed from the full-length cDNA sequences of *Fragaria* × *ananassa* corresponding to each accession number obtained from the GenBank [34]; ^2^ Gene names: *BG2-1*, β-1,3-glucanase 2-1; *BG2-2*, β-1,3-glucanase 2-2; *BG2-3*, β-1,3-glucanase 2-3; *PGIP1*, polygalacturonase inhibiting protein 1; *PGIP2*, polygalacturonase inhibiting protein 2; *CHI2-2*, chitinase 2-2; *CHI3-1*, chitinase 3-1; ^3^ Protein family names and gene IDs or gene names according to Amil-Ruiz et al. [13].

## Supplementary Materials

Supplementary materials can be found at www.mdpi.com/1422-0067/18/7/1420/s1.

## Abbreviations

BG	β-1,3-glucanase
CHI	Chitinase
CTAB	Cetyltrimethylammonium bromide
GAPDH	Glyceraldehyde 3-phosphate dehydrogenase
JA	Jasmonic acid
JAs	Jasmonates
ISR	Induced systemic resistance
MAPK	Mitogen-activated protein kinase
MeJA	Methyl jasmonate
PDA	Potato dextrose agar
PGIP	Polygalacturonase-inhibiting protein
PR	Pathogenesis-related protein
SAR	Systemic acquired resistance
SSC	Soluble solids concentration
SDW	Sterile distilled water
RT-qPCR	Quantitative reverse transcription PCR
TA	Titratable acidity
