# Supplementary material for: Independent Preharvest Applications of Methyl Jasmonate and Chitosan Elicit Differential Upregulation of Defense-Related Genes with Reduced Incidence of Gray Mold Decay during Postharvest Storage of Fragaria chiloensis Fruit
Source: Int J Mol Sci. 2017 Jul 3;18(7):1420. doi: 10.3390/ijms18071420 (PMC5535912; doi:10.3390/ijms18071420)
Supplement: Supplementary file 1 [file ijms-18-01420-s001.pdf]

Supplementary Materials: Independent Preharvest Applications of Methyl Jasmonate and Chitosan Elicit Differential Upregulation of Defense-Related Genes with Reduced Incidence of Gray Mold Decay during Postharvest Storage of *Fragaria chiloensis* Fruit

Table S1. Changes in fruit firmness (N), weight (g), and color parameters from control-, chitosan-, and MeJA-treated *Fragaria chiloensis* fruit at 0 and 72 h of postharvest storage.

Postharvest storage (h)	Treatment	Firmness (N)[1]	Weight (g)	Color parameters[2]				
				*L**	*a**	*b**	Chroma	Hue (h^o)
0	Control	7.79 ± 0.69 a[3]	1.50 ± 0.33 a	58.68 ± 3.20 a	18.27 ± 5.08 a	18.85 ± 2.04 a	27.36 ± 2.93 a	49.06 ± 8.94 a
	Chitosan	6.91 ± 0.37 a	1.58 ± 0.31 a	61.21 ± 3.94 a	17.63 ± 3.70 a	18.48 ± 1.89 a	26.39 ± 2.83 a	48.86 ± 6.71 a
	MeJA	8.45 ± 0.63 a	1.55 ± 0.13 a	58.59 ± 3.61 a	17.15 ± 4.49 a	17.49 ± 2.31 a	25.52 ± 2.68 a	48.75 ± 9.14 a
72	Control	9.66 ± 0.46 b	1.59 ± 0.45 a	56.13 ± 3.94 a	18.86 ± 3.56 a	18.10 ± 2.23 a	26.96 ± 2.64 a	46.60 ± 7.35 a
	Chitosan	12.16 ± 1.43 a	1.64 ± 0.29 a	55.02 ± 2.77 ab	18.20 ± 4.58 a	18.58 ± 2.26 a	28.05 ± 6.39 a	49.41 ± 7.74 a
	MeJA	9.73 ± 1.15 b	1.40 ± 0.15 a	52.99 ± 3.98 b	17.22 ± 6.06 a	18.48 ± 1.67 a	26.46 ± 3.32 a	50.23 ± 11.70 a

[1] Fruit firmness was expressed in Newton (N).

[2] Skin color was expressed as CIELAB scale (*L**, *a**, *b**) along with the dimensions of color chroma and hue angle (h^o).

[3] Data were analyzed by ANOVA test and differences among means ± SD (n = 12) were determined using the Tukey test. Different letters indicate significant differences between treatments ($p < 0.05$) for each hour. For experimental details see the Materials and Methods Section.

Table S2. Relative expression values of *PR* and *PGIP* genes evaluated in different treatments (i.e., control, chitosan and MeJA) and in –*Bc* and +*Bc* fruit. Data were analyzed by ANOVA test and differences between means ± SE (n = 3) were determined using the Tukey test.

Gene	Hours post inoculation (hpi)	Treatment and inoculation					
		Control		Chitosan		MeJA	
		–*Bc*	+*Bc*	–*Bc*	+*Bc*	–*Bc*	+*Bc*
FcBG2-1	0	1.04 ± 0.19 b[1]	1.04 ± 0.19 b	14.54 ± 2.15 b	14.54 ± 2.15 a	10.96 ± 0.80 ab	10.96 ± 0.80 c
	2	1.53 ± 0.45 b	1.25 ± 0.09 b	14.42 ± 1.89 b	18.47 ± 2.64 a	6.43 ± 2.80 b	5.78 ± 1.00 c
	24	7.17 ± 0.85 ab	2.78 ± 0.38 b	11.13 ± 0.56 b	18.28 ± 2.31 a	12.68 ± 3.14 ab	26.37 ± 2.34 b
	48	23.60 ± 5.20 a	28.52 ± 1.46 a	37.59 ± 8.77 a	21.99 ± 2.66 a	27.63 ± 3.01 a	41.44 ± 2.34 a
	72	11.13 ± 2.51 ab	9.02 ± 2.50 b	50.55 ± 3.57 a	20.56 ± 0.46 a	29.80 ± 1.05 a	28.73 ± 1.24 b
FcBG2-2	0	1.01 ± 0.08 a	1.01 ± 0.08 a	6.24 ± 0.26 b	6.24 ± 0.26 b	8.53 ± 3.95 bc	8.53 ± 3.95 bc
	2	1.30 ± 0.15 a	1.26 ± 0.07 a	7.20 ± 0.59 b	7.50 ± 0.25 b	2.98 ± 0.22 c	3.36 ± 1.61 c
	24	7.48 ± 0.33 a	1.28 ± 0.23 a	10.85 ± 6.62 b	18.02 ± 2.64 a	16.79 ± 1.59 b	11.83 ± 0.79 b
	48	9.73 ± 1.84 a	8.10 ± 0.96 a	14.96 ± 4.10 b	10.59 ± 1.15 ab	17.11 ± 2.64 b	23.03 ± 0.81 a
	72	6.75 ± 1.42 a	6.64 ± 1.09 a	48.88 ± 0.93 a	14.44 ± 0.37 ab	29.71 ± 1.78 a	20.80 ± 2.90 a
FcBG2-3	0	1.00 ± 0.08 a	1.00 ± 0.08 b	6.24 ± 0.26 c	6.24 ± 0.26 b	8.53 ± 3.95 c	8.53 ± 3.95 bc
	2	1.15 ± 0.10 a	1.00 ± 0.17 b	8.25 ± 0.20 c	7.73 ± 0.95 b	3.33 ± 0.08 c	4.88 ± 2.37 c
	24	6.61 ± 0.57 a	1.59 ± 0.20 b	11.63 ± 8.60 bc	16.42 ± 0.67 ab	8.66 ± 2.51 bc	14.84 ± 0.45 b
	48	15.11 ± 3.11 a	12.38 ± 0.96 a	23.77 ± 4.04 b	11.54 ± 0.26 ab	22.77 ± 2.70 ab	32.84 ± 3.54 a
	72	8.12 ± 1.13 a	3.36 ± 0.27 ab	53.37 ± 18.91 a	19.79 ± 1.15 a	23.53 ± 3.55 a	28.02 ± 4.95 a
FcCHI2-2	0	1.02 ± 0.14 a	1.02 ± 0.14 bc	1.98 ± 0.35 a	1.98 ± 0.35 b	1.84 ± 0.16 a	1.84 ± 0.16 b
	2	1.63 ± 0.27 a	1.91 ± 0.25 b	1.38 ± 0.40 a	2.01 ± 0.03 b	3.31 ± 0.29 a	2.63 ± 0.32 b
	24	2.65 ± 0.44 a	1.30 ± 0.09 bc	4.40 ± 3.29 a	2.44 ± 0.51 b	2.56 ± 1.71 a	1.28 ± 0.11 b
	48	2.86 ± 0.25 a	3.82 ± 0.27 a	3.31 ± 0.90 a	0.22 ± 0.05 c	5.14 ± 0.75 a	6.80 ± 0.36 a
	72	0.33 ± 0.03 a	0.32 ± 0.08 c	1.88 ± 0.33 a	9.23 ± 0.22 a	1.78 ± 1.08 a	6.29 ± 0.61 a
FcCHI3-1	0	1.08 ± 0.28 a	1.08 ± 0.28 a	0.60 ± 0.10 a	0.60 ± 0.10 b	1.24 ± 0.55 a	1.24 ± 0.55 a
	2	1.62 ± 0.56 a	1.23 ± 0.17 a	0.54 ± 0.12 a	0.58 ± 0.24 b	1.39 ± 0.24 a	0.28 ± 0.04 a
	24	1.02 ± 0.33 a	0.18 ± 0.03 a	0.32 ± 0.0015 a	0.79 ± 0.03 ab	0.93 ± 0.42 a	0.19 ± 0.02 a
	48	0.24 ± 0.04 a	0.39 ± 0.07 a	0.62 ± 0.28 a	0.21 ± 0.04 b	0.66 ± 0.13 a	0.62 ± 0.14 a
	72	0.34 ± 0.09 a	0.68 ± 0.19 a	1.40 ± 0.57 a	1.98 ± 0.55 a	0.77 ± 0.20 a	0.66 ± 0.10 a
FcPGIP1	0	1.03 ± 0.19 a	1.03 ± 0.19 b	23.58 ± 2.31 b	23.58 ± 2.31 a	11.26 ± 1.15 a	11.26 ± 1.15 b
	2	1.29 ± 0.19 a	1.36 ± 0.21 b	39.73 ± 3.87 a	35.34 ± 4.46 a	6.29 ± 0.85 a	6.18 ± 1.32 b
	24	2.15 ± 0.15 a	9.90 ± 0.20 ab	3.58 ± 0.31 c	39.98 ± 9.13 a	8.38 ± 0.20 a	10.95 ± 1.03 b
	48	7.18 ± 3.84 a	21.13 ± 2.99 a	5.28 ± 1.41 c	3.55 ± 0.55 b	7.06 ± 3.29 a	7.29 ± 1.36 b
	72	4.61 ± 0.86 a	1.48 ± 0.01 ab	20.66 ± 1.88 b	5.37 ± 0.44 b	3.38 ± 0.71 a	41.15 ± 5.44 a
FcPGIP2	0	1.00 ± 0.04 a	1.00 ± 0.04 a	17.55 ± 1.60 ab	17.55 ± 1.60 b	10.59 ± 1.03 a	10.59 ± 1.03 a
	2	1.31 ± 0.31 a	1.39 ± 0.19 a	19.31 ± 1.57 a	25.86 ± 1.31 a	5.11 ± 0.35 ab	4.11 ± 0.92 b
	24	2.30 ± 0.11 a	5.56 ± 1.01 a	3.18 ± 0.41 c	26.25 ± 1.97 a	1.41 ± 0.28 b	9.16 ± 1.81 a
	48	1.96 ± 0.48 a	2.75 ± 0.15 a	2.95 ± 0.61 c	11.69 ± 2.15 b	6.42 ± 0.14 ab	3.05 ± 0.13 b
	72	3.98 ± 0.72 a	1.24 ± 0.07 a	10.72 ± 5.47 bc	5.26 ± 0.71 c	2.60 ± 0.06 ab	2.96 ± 0.52 b

[1] Different letters indicate significant differences between hpi ($p < 0.05$) for each treatment and –*Bc*/+*Bc* fruit in each gene. For experimental details see the Materials and Methods Section.

Table S3. Differences between means of relative expression values of –*Bc* and +*Bc* fruit. Numbers and asterisks in blue and red indicate fold reduction or induction in +*Bc* fruit with respect to –*Bc* fruit, respectively.

Gene	Hours post inoculation (hpi)	Treatments								
		Control			Chitosan			MeJA		
		Mean diff.[1]	Significant?[2]	P-value	Mean diff.	Significant?	P-value	Mean diff.	Significant?	P-value
FcBG2-1	0	0	ns	>0.9999	0	ns	>0.9999	0	ns	>0.9999
	2	–0.2748	ns	>0.9999	4.044	ns	>0.9999	–0.6481	ns	>0.9999
	24	–4.398	ns	>0.9999	7.147	ns	0.9819	13.69	ns	0.1067
	48	4.916	ns	>0.9999	–15.6	*	0.0265	14.41	ns	0.065
	72	–2.114	ns	>0.9999	–41.53	****	<0.0001	–1.073	ns	>0.9999
FcBG2-2	0	0	ns	>0.9999	0	ns	>0.9999	0	ns	>0.9999
	2	–0.0428	ns	>0.9999	–5.94	ns	0.9461	0.3746	ns	>0.9999
	24	–6.194	ns	0.9188	–9.568	ns	0.1975	–4.961	ns	0.9939
	48	–1.634	ns	>0.9999	–6.869	ns	0.8077	5.92	ns	0.948
	72	–0.1151	ns	>0.9999	–42.24	****	<0.0001	–8.911	ns	0.3161
FcBG2-3	0	0	ns	>0.9999	0	ns	>0.9999	0	ns	>0.9999
	2	–0.1535	ns	>0.9999	–0.5219	ns	>0.9999	1.554	ns	>0.9999
	24	–5.015	ns	0.9994	4.796	ns	0.9997	6.183	ns	0.987
	48	–2.73	ns	>0.9999	–12.23	ns	0.1004	10.07	ns	0.3962
	72	–4.762	ns	0.9998	–33.58	****	<0.0001	4.496	ns	>0.9999
FcCHI2-2	0	0	ns	>0.9999	0	ns	>0.9999	0	ns	>0.9999
	2	0.2809	ns	>0.9999	0.6333	ns	>0.9999	–0.5646	ns	>0.9999
	24	–1.348	ns	>0.9999	–1.958	ns	0.9932	–1.284	ns	>0.9999
	48	0.9638	ns	>0.9999	–3.093	ns	0.5587	1.666	ns	0.9994
	72	–0.008533	ns	>0.9999	7.354	****	<0.0001	4.514	*	0.0335
FcCHI3-1	0	0	ns	>0.9999	0	ns	>0.9999	0	ns	>0.9999
	2	—0.3911	ns	>0.9999	0.04217	ns	>0.9999	1.106	ns	0.7854
	24	–0.8329	ns	0.9519	0.3635	ns	>0.9999	–0.7386	ns	0.9882
	48	0.1448	ns	>0.9999	–0.4121	ns	>0.9999	–0.0384	ns	>0.9999
	72	0.3441	ns	>0.9999	0.5881	ns	>0.9999	–0.1065	ns	>0.9999
FcPGIP1	0	0	ns	>0.9999	0	ns	>0.9999	0	ns	>0.9999
	2	0.0694	ns	>0.9999	–4.394	ns	>0.9999	–0.1038	ns	>0.9999
	24	7.747	ns	0.9803	36.4	****	<0.0001	2.572	ns	>0.9999
	48	13.95	ns	0.168	–1.732	ns	>0.9999	0.2281	ns	>0.9999
	72	–3.208	ns	>0.9999	–14.86	ns	0.0975	37.66	****	<0.0001
FcPGIP2	0	0	ns	>0.9999	0	ns	>0.9999	0	ns	>0.9999
	2	0.08213	ns	>0.9999	6.543	ns	0.2289	1	ns	>0.9999
	24	3.257	ns	0.9974	23.08	****	<0.0001	7.746	ns	0.0518
	48	0.7858	ns	>0.9999	8.741	*	0.0117	–3.367	ns	0.9958
	72	–2.738	ns	0.9999	–5.46	ns	0.5788	0.3587	ns	>0.9999

[1] Multiple comparison analysis between means was performed using two-way ANOVA and differences between means were determined using the Tukey test.

[2] ns, not significant; *, $P < 0.05$; **, $P < 0.01$; ***, $P < 0.001$; ****, $P < 0.0001$.

Table S4. Upregulation values for *PR* and *PGIP* genes in chitosan- and MeJA-treated fruit with respect to control treatment with –*Bc* and +*Bc* inoculations (fold-change statistically significant in red, $p < 0.05$).

Gene	Hours post inoculation (hpi)	Treatment and inoculation			
		Chitosan		MeJA	
		**–*Bc***	**+*Bc***	**–*Bc***	**+*Bc***
FcBG2-1	0	14.0	14.0	10.6	10.6
	2	9.5	14.8	4.2	4.6
	24	1.6	6.6	1.8	9.5
	48	1.6	0.8	1.1	1.5
	72	4.5	2.3	2.7	3.2
FcBG2-2	0	6.2	6.2	8.5	8.5
	2	5.5	6.0	2.3	2.7
	24	1.5	14.0	2.2	9.2
	48	1.5	1.3	1.8	2.8
	72	7.2	2.2	4.4	3.1
FcBG2-3	0	8.1	8.1	6.7	6.7
	2	7.2	7.7	2.9	4.9
	24	1.8	10.3	1.3	9.3
	48	1.6	0.9	1.5	2.7
	72	6.6	5.9	2.9	8.3
FcCHI2-2	0	1.9	1.9	1.8	1.8
	2	0.8	1.1	2.0	1.4
	24	1.7	1.9	1.0	1.0
	48	1.2	0.1	1.8	1.8
	72	5.7	28.6	5.4	19.5
FcCHI3-1	0	0.6	0.6	1.1	1.1
	2	0.3	0.5	0.9	2.0
	24	0.2	4.3	0.9	1.0
	48	2.6	0.5	2.7	1.6
	72	4.2	2.9	1.5	1.0
FcPGIP1	0	22.9	22.9	10.9	10.9
	2	30.8	26.0	4.9	4.6
	24	1.7	4.0	3.9	1.1
	48	0.7	0.2	1.0	0.3
	72	4.5	3.6	0.7	27.7
FcPGIP2	0	17.5	17.5	10.6	10.6
	2	14.7	18.6	3.9	2.9
	24	1.4	4.7	0.6	1.6
	48	1.5	4.3	3.3	1.1
	72	2.7	4.2	0.7	2.4
